# Uric acid-to-HDL-cholesterol ratio and incident carotid atherosclerosis: a retrospective cohort study

**DOI:** 10.3389/fnut.2026.1951708

**Published:** 2026-10-15

**Authors:** Hezhi Wen, Kaiyang Shao, Yaling Li, Ruyi Cai, Ting Sun

**Affiliations:** Department of Health Management Center, Second Affiliated Hospital, School of Medicine, Zhejiang University, Hangzhou, Zhejiang, China

**Keywords:** cardiovascular disease, carotid atherosclerosis, retrospective cohort, risk stratification, uric acid-to-HDL-cholesterol ratio

## Abstract

**Background:**

The uric acid-to-HDL-cholesterol ratio (UHR) has been proposed as a composite cardiometabolic biomarker, but prospective evidence linking UHR to incident carotid atherosclerosis remains limited.

**Methods:**

In a retrospective cohort of 16,572 adults from a tertiary health examination center in Zhejiang, China, we examined the association between baseline UHR and incident carotid artery lesions using Cox proportional hazards regression. Incremental predictive performance was evaluated using C-statistic, integrated discrimination improvement, continuous net reclassification improvement, median improvement in individual predicted event probabilities, and decision curve analysis.

**Results:**

Over a mean follow-up of 2.7 years, 4,454 incident events occurred. Each 1-SD increment in baseline UHR was independently associated with a 6% higher hazard of incident carotid artery lesions (HR 1.06, 95% CI 1.01–1.11; *p* = 0.015). In exploratory sex-stratified analyses, the association was statistically significant in males (HR 1.07, 95% CI 1.02–1.12; *p* = 0.010) but not in females (HR 0.98, 95% CI 0.93–1.04; *p* = 0.524), though the formal interaction test was not significant (*p* = 0.261). UHR did not improve discrimination (ΔC = 0.0003) or net clinical benefit despite significant changes in individual predicted event probabilities in males (*p* = 0.008).

**Conclusion:**

Higher baseline UHR was independently associated with short-term carotid atherosclerotic progression. Exploratory analyses suggested a possible sex difference, but this requires confirmation in adequately powered studies. While UHR provides independent prognostic information, it does not meaningfully improve risk stratification beyond traditional cardiovascular risk factors in health examination populations.

## 1 Introduction

Cardiovascular disease (CVD) remains a leading cause of mortality worldwide and represents a growing public health burden in ageing populations, including those in China (1). Atherosclerosis, a chronic inflammatory process characterized by progressive lipid deposition and fibrous tissue accumulation within the arterial wall, underlies the majority of CVD events (2). Established risk factors such as hypertension, dyslipidemia, diabetes, obesity, and smoking contribute to endothelial dysfunction and oxidative stress, which drive atherosclerotic progression (3, 4). The carotid artery is one of the most common sites of early atherosclerotic involvement, and carotid atherosclerosis (CAS), manifested as increased carotid intima-media thickness (cIMT) and plaque formation, has become a well-recognized indicator of systemic vascular risk (5, 6). Large-scale epidemiological data from China indicate that CAS affects a considerable proportion of the general adult population, with the burden disproportionately concentrated among those with cardiometabolic risk factors (7). Identifying reliable biomarkers for early risk stratification therefore remains a priority in cardiovascular prevention.

Uric acid (UA), the terminal product of purine metabolism, promotes atherogenesis through multiple pathways, including oxidative stress, NLRP3 inflammasome activation, endothelial dysfunction, and vascular smooth muscle cell proliferation (8, 9). High-density lipoprotein cholesterol (HDL-C) counteracts these processes via reverse cholesterol transport, antioxidant activity, and anti-inflammatory signaling, and reduced HDL-C has been independently associated with increased cardiovascular risk (10, 11). Because UA and HDL-C have opposing effects on vascular health, the UA-to-HDL-C ratio (UHR) may serve as a composite biomarker reflecting the balance between oxidative-inflammatory burden and lipoprotein-mediated vascular protection. Associations between elevated UHR and cardiometabolic risk have been reported across a range of conditions, including metabolic syndrome, type 2 diabetes mellitus, nonalcoholic fatty liver disease, and coronary artery disease (12–16).

Recent cross-sectional studies have documented independent associations between elevated UHR and carotid plaque in health examination cohorts and in patients with type 2 diabetes, and UHR showed better discriminative performance than UA or HDL-C alone in receiver operating characteristic (ROC) analyses. However, these designs cannot establish temporality or distinguish prevalent from incident disease, and prospective evidence using time-to-event analysis with rigorous adjustment for confounding remains limited.

To address this gap, the present study used retrospective cohort data from a general health examination population to investigate the association of baseline UHR with incident CAS risk. We further examined whether distinct longitudinal UHR trajectory patterns, identified by latent class growth analysis, were independently associated with CAS incidence, given that a single baseline measurement may not adequately reflect the cumulative atherogenic exposure associated with sustained UHR elevation over time.

## 2 Methods

### 2.1 Study design and population

This retrospective cohort study used data from the Health Management Center of a Grade A tertiary hospital in Zhejiang, China. Participants who underwent at least two consecutive annual health examinations between 2022 and 2025 were considered for inclusion. Eligibility criteria were: (1) age ≥ 18 years at baseline; (2) complete baseline measurements of serum UA and HDL-C; and (3) no evidence of carotid artery lesions at baseline ultrasonography. Participants were excluded if they had a prior diagnosis of coronary artery disease, stroke, or peripheral artery disease; active malignancy; end-stage renal disease; or missing data for any of the primary covariates included in multivariable analyses (age, sex, BMI, SBP, DBP, FBG, TG, TC, creatinine, or eGFR). The study flowchart detailing participant selection is shown in Figure 1. The study was approved by the Human Research Ethics Committee of the Second Affiliated Hospital of Zhejiang University School of Medicine (approval number: 2026LSYD1424) and conducted in accordance with the Declaration of Helsinki. The requirement for written informed consent was waived by the ethics committee owing to the retrospective use of anonymized routine health examination data.

**Figure 1:**
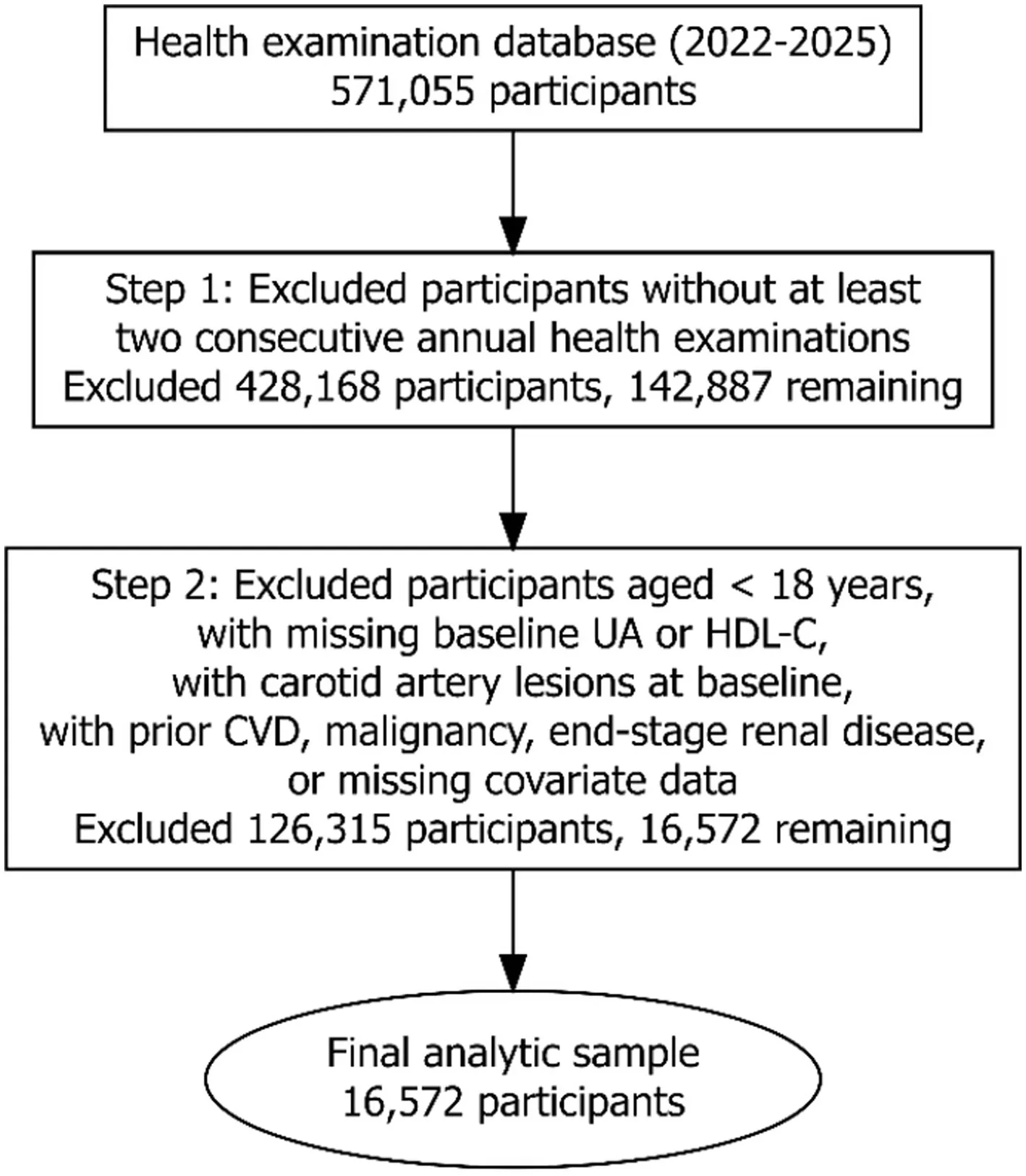
Flowchart.

### 2.2 Exposure: UHR calculation

The primary exposure was the UA-to-HDL-C ratio (UHR), calculated as serum UA (μmol/L) divided by HDL-C (mmol/L) at baseline. Venous blood samples were drawn after a minimum 8-h overnight fast. Serum UA was measured by the uricase-peroxidase enzymatic method, and HDL-C by direct homogeneous enzymatic colorimetric assay, both performed on an automated biochemical analyzer in a single accredited laboratory using consistent instrumentation throughout the study period.

### 2.3 Outcome definition

The primary outcome was incident carotid artery lesions, defined as the first detection during follow-up of cIMT thickening (1.0 mm ≤ cIMT < 1.5 mm) or carotid plaque, assessed by carotid color Doppler ultrasonography. Carotid plaque was defined as cIMT ≥ 1.5 mm, or a focal protrusion into the arterial lumen with a thickness ≥ 0.5 mm or exceeding 50% of the surrounding cIMT.

Trained and certified sonographers performed all ultrasound examinations. cIMT measurements and plaque assessments were performed manually using electronic calipers on B-mode ultrasound images, following the standardized protocol based on the Mannheim Carotid Intima-Media Thickness and Plaque Consensus (17). All sonographers completed institutional standardized training to maintain measurement consistency.

Follow-up began at the date of the baseline examination and continued until the first occurrence of carotid artery lesions, death, loss to follow-up, or end of the study period, whichever occurred first.

### 2.4 Covariates

Covariates included age, sex, body mass index (BMI; kg/m^2^), systolic blood pressure (SBP), diastolic blood pressure (DBP), fasting blood glucose (FBG), triglycerides (TG), total cholesterol (TC), serum creatinine, and eGFR, selected based on established cardiovascular risk factors and clinical relevance. eGFR was calculated using the CKD-EPI 2021 creatinine equation. All biochemical variables were measured from the same fasting venous blood samples.

### 2.5 Longitudinal trajectory analysis

Longitudinal UHR trajectories were identified using latent class growth analysis (LCGA) applied to annual UHR measurements at each health examination visit, with time modeled as years since baseline. The optimal number of trajectory groups was determined by a combination of statistical and practical criteria: minimizing the Bayesian Information Criterion (BIC), ensuring a minimum class size of 5% of the total cohort, and achieving average posterior probabilities of group membership exceeding 0.70. Models comprising two to five trajectory groups were evaluated, and the three-group solution was selected based on these criteria and clinical interpretability. Analyses were performed using the lcmm package in R.

### 2.6 Statistical analysis

Baseline UHR was the primary exposure in all Cox regression models, modeled per 1-standard deviation (SD) increment (SD calculated in the overall study population); UHR values from subsequent annual examinations were used solely for trajectory analyses. Prior to continuous analyses, UHR values were Winsorized at the 1st and 99th percentiles. Low-density lipoprotein cholesterol (LDL-C) was measured and presented descriptively in Table 1 but excluded from regression models owing to its structural collinearity with TC, consistent with the Friedewald equation.

**Table 1:** Baseline characteristics of study participants by UHR longitudinal trajectory group.

Characteristic	Overall (*n* = 16,572)	Low UHR (*n* = 8,561)	Moderate UHR (*n* = 6,020)	High UHR (*n* = 1991)	*p-*value
Age, years	42.78 (10.47)	42.97 (10.51)	43.15 (10.60)	40.86 (9.63)	<0.001
BMI, kg/m^2^	23.49 (3.30)	21.86 (2.62)	24.76 (2.89)	26.64 (3.11)	<0.001
SBP, mmHg	118.51 (15.45)	114.61 (15.03)	122.00 (14.82)	124.65 (14.55)	<0.001
DBP, mmHg	72.04 (10.73)	69.17 (10.08)	74.44 (10.47)	77.11 (10.54)	<0.001
FBG, mmol/L	5.15 (0.80)	5.03 (0.69)	5.26 (0.86)	5.35 (0.99)	<0.001
TG, mmol/L	1.16 [0.82, 1.72]	0.89 [0.70, 1.21]	1.42 [1.05, 1.99]	2.19 [1.60, 3.16]	<0.001
TC, mmol/L	5.11 (0.95)	5.14 (0.95)	5.08 (0.94)	5.10 (0.96)	0.001
LDL-C, mmol/L	2.89 (0.75)	2.77 (0.74)	3.01 (0.73)	3.04 (0.72)	<0.001
HDL-C, mmol/L	1.37 (0.36)	1.60 (0.31)	1.18 (0.20)	0.93 (0.18)	<0.001
UA, μmol/L	335.00 [275.00, 400.00]	279.00 [244.00, 319.00]	381.00 [344.00, 422.00]	461.00 [420.00, 510.00]	<0.001
UHR	251.72 [179.41, 347.27]	181.88 [147.76, 217.90]	325.40 [285.07, 371.30]	488.61 [439.35, 561.92]	<0.001
Creatinine, μmol/L	69.50 [59.10, 80.80]	61.20 [55.00, 69.40]	77.40 [68.90, 85.10]	81.10 [73.90, 88.40]	<0.001
eGFR, mL/min/1.73m^2^	104.55 [95.15, 112.88]	106.46 [97.50, 114.61]	102.51 [93.30, 110.85]	102.18 [91.72, 110.80]	<0.001
Follow-up, years	2.71 (0.67)	2.74 (0.64)	2.67 (0.70)	2.69 (0.69)	<0.001
Sex, *n* (%)					<0.001
Male	8,544 (51.6)	1734 (20.3)	4,880 (81.1)	1930 (96.9)	
Female	8,028 (48.4)	6,827 (79.7)	1,140 (18.9)	61 (3.1)	
Incident carotid artery lesions, *n* (%)					<0.001
No	12,118 (73.1)	6,470 (75.6)	4,213 (70.0)	1,435 (72.1)	
Yes	4,454 (26.9)	2091 (24.4)	1807 (30.0)	556 (27.9)	

Values are presented as mean (SD), median [IQR], or *n* (%), as appropriate. UHR, uric acid-to-HDL-C ratio; BMI, body mass index; SBP, systolic blood pressure; DBP, diastolic blood pressure; FBG, fasting blood glucose; TG, triglycerides; TC, total cholesterol; LDL-C, low-density lipoprotein cholesterol; HDL-C, high-density lipoprotein cholesterol; UA, uric acid; eGFR, estimated glomerular filtration rate. LDL-C is presented for descriptive purposes only and was not included in regression analyses due to collinearity with TC. *p*-values were calculated using one-way ANOVA, Kruskal–Wallis test, or chi-square test, as appropriate.

Continuous variables are presented as mean (SD) or median (interquartile range [IQR]) according to distributional normality (Kolmogorov–Smirnov test); categorical variables as frequencies and percentages. Between-group comparisons used one-way analysis of variance (ANOVA), Kruskal–Wallis test, or chi-square test, as appropriate.

The association between baseline UHR and incident carotid artery lesions was assessed using Cox proportional hazards regression across three sequential models: Model 1 (unadjusted); Model 2 (adjusted for age and sex); and Model 3 (fully adjusted for age, sex, BMI, SBP, DBP, FBG, TG, TC, creatinine, and eGFR). The proportional hazards assumption was verified using Schoenfeld residuals. Dose–response relationships were evaluated using restricted cubic spline (RCS) Cox regression with four knots at the 5th, 35th, 65th, and 95th percentiles of the UHR distribution, with the median as reference. Sex-stratified Cox and RCS analyses were conducted given established sex differences in uric acid metabolism; effect modification by sex was assessed by likelihood ratio test. Associations between UHR trajectory group membership and incident carotid artery lesions were examined using the same three Cox models, with the Low UHR group as reference.

The incremental predictive value of UHR beyond traditional risk factors was evaluated by comparing a base model (age, sex, BMI, SBP, DBP, FBG, TG, TC, creatinine, and eGFR) with an extended model additionally including baseline UHR, using the C-statistic (ΔC), integrated discrimination improvement (IDI), continuous net reclassification improvement (NRI), and median improvement in individual predicted event probabilities (MED), estimated at the median event time via the survIDINRI package. Given the sex-specific association observed in primary analyses, this analysis was additionally conducted in the male subgroup.

Decision curve analysis (DCA) was performed at a 2-year prediction horizon to compare the clinical net benefit of the base and UHR-added models across a range of threshold probabilities.

All analyses were performed in R version 4.5.1. Two-sided *p* < 0.05 was considered statistically significant.

## 3 Results

### 3.1 Baseline characteristics

A total of 16,572 participants were included in the final analysis. Latent class growth analysis identified three distinct UHR trajectory groups across the follow-up period: Low UHR (*n* = 8,561, 51.7%), Moderate UHR (*n* = 6,020, 36.3%), and High UHR (*n* = 1,991, 12.0%). As shown in Figure 2, mean UHR in all three groups showed a general declining trend over the four annual assessment time points, while maintaining stable between-group separation throughout follow-up.

**Figure 2:**
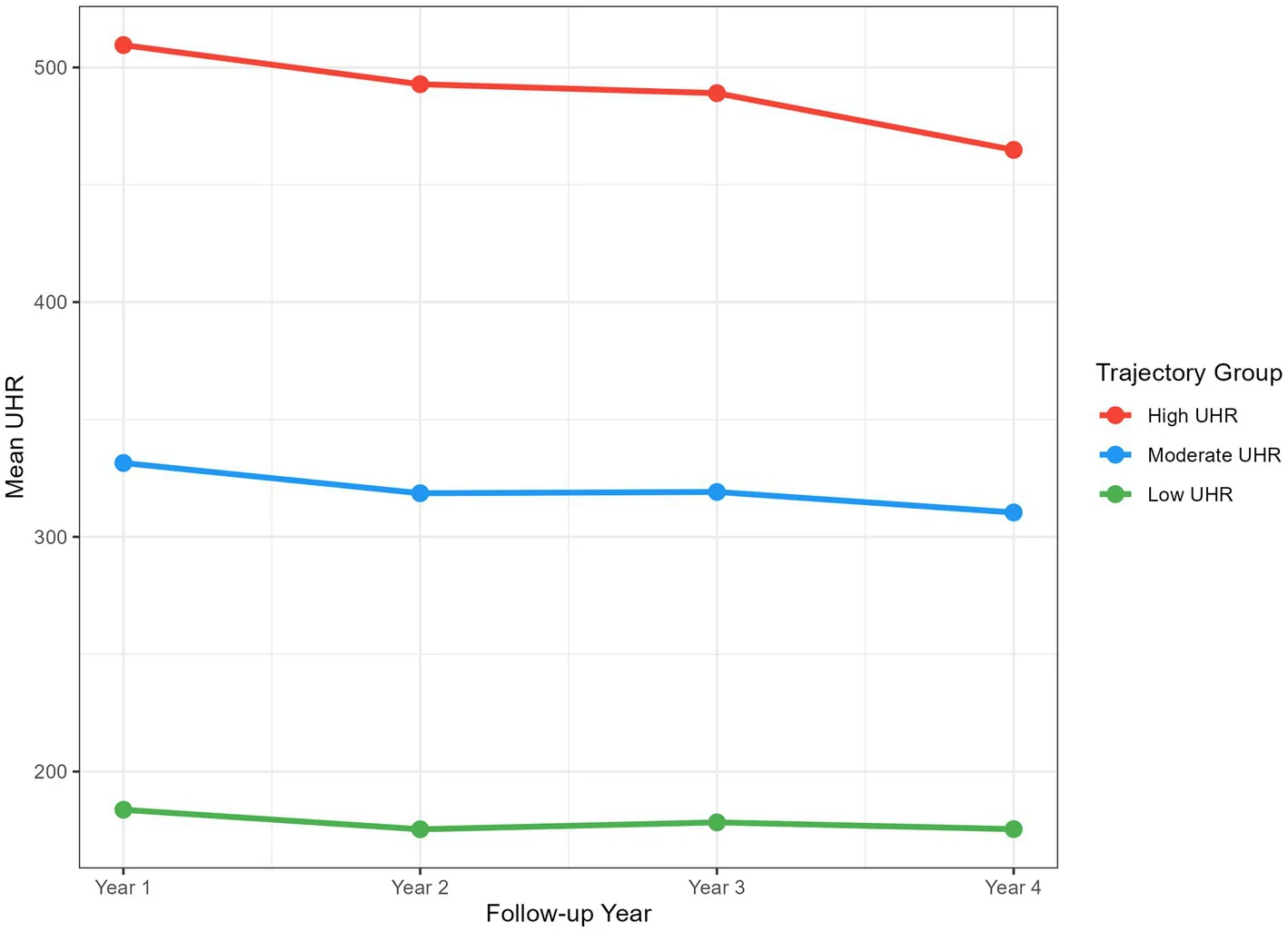
Longitudinal trajectories of UHR identified by latent class growth analysis. Lines represent mean UHR values at each annual assessment for the three trajectory groups.

Baseline characteristics stratified by trajectory group are presented in Table 1. Significant differences across groups were observed for all metabolic parameters, including BMI, systolic and diastolic blood pressure, fasting blood glucose, triglycerides, HDL-C, serum uric acid, creatinine, and eGFR (all *p* < 0.001). The High UHR group exhibited the highest values for BMI, blood pressure, FBG, TG, UA, and creatinine, and the lowest HDL-C and eGFR. Although total cholesterol differed statistically across groups (*p* = 0.001), the absolute differences were clinically negligible (5.14, 5.08, and 5.10 mmol/L). A marked difference in sex distribution was observed: male participants comprised 20.3% of the Low UHR group, 81.1% of the Moderate group, and 96.9% of the High UHR group (*p* < 0.001). Mean (SD) follow-up duration was 2.74 (0.64), 2.67 (0.70), and 2.69 (0.69) years in the Low, Moderate, and High UHR groups, respectively (*p* < 0.001). Incident carotid artery lesions occurred in 2,091 (24.4%), 1,807 (30.0%), and 556 (27.9%) participants in the Low, Moderate, and High UHR groups, respectively.

### 3.2 Association between baseline UHR and incident carotid artery lesions

Results from Cox proportional hazards regression are presented in Table 2. In the unadjusted model (Model 1), each 1-SD increment in baseline UHR was associated with a significantly higher hazard of incident carotid artery lesions (HR 1.09, 95% CI 1.06–1.12; *p* < 0.001), with significant evidence of non-linearity (*P* non-linear < 0.001).

**Table 2:** Association between baseline UHR and incident carotid artery lesions.

Model	HR (95% CI)	*p*-value	*P* overall	*P* non-linear
Model 1	1.09 (1.06–1.12)	<0.001	<0.001	<0.001
Model 2	1.05 (1.02–1.09)	0.005	0.015	0.243
Model 3	1.06 (1.01–1.11)	0.015	0.046	0.346

HR, hazard ratio; CI, confidence interval. Model 1: unadjusted. Model 2: adjusted for age and sex. Model 3: additionally adjusted for BMI, SBP, DBP, FBG, TG, TC, creatinine, and eGFR. HDL-C and UA were not included as covariates as they constitute the components of UHR; LDL-C was excluded due to collinearity with TC. HR expressed per 1-SD increment in UHR (1 SD = 123.35 units, calculated in the overall study population). P value refers to the Wald test P value for the HR per 1-SD increment; P overall and P nonlinear refer to the overall and nonlinearity tests from restricted cubic spline (RCS) Cox regression with 4 knots at the 5th, 35th, 65th, and 95th percentiles of the UHR distribution (121.47, 205.82, 304.67, and 512.41 units); reference set at the median UHR (251.93 units, derived from Winsorized values used in regression analyses).

The crude spline exhibited an inverted U-shaped pattern with a prominent decline at higher UHR values, attributable to confounding by sex (Supplementary Figure 1). After adjustment for age and sex (Model 2), the hazard ratio attenuated to 1.05 (95% CI 1.02–1.09, *p* = 0.005), and the non-linearity was no longer significant (*P* non-linear = 0.243). In the fully adjusted model incorporating age, sex, BMI, SBP, DBP, FBG, TG, TC, creatinine, and eGFR (Model 3), a statistically significant association was retained (HR 1.06, 95% CI 1.01–1.11; *p* = 0.015), with no significant departure from linearity (*P* non-linear = 0.346). The evolution of the RCS spline across the three models is illustrated in Supplementary Figure 1.

### 3.3 Dose–response relationship

Unlike the J- or U-shaped patterns reported for serum uric acid alone in clinical populations with established cardiovascular disease, the adjusted dose–response relationship in the present cohort differed. The association showed a modest protective trend at low UHR values, followed by a progressive increase above the median. This pattern is consistent with a threshold effect rather than a J- or U-shaped association. The dose–response relationship between baseline UHR and incident carotid artery lesions in the fully adjusted model is shown in Figure 3. The overall association was statistically significant (*P* overall = 0.046), with no significant departure from linearity (*P* non-linear = 0.346).

**Figure 3:**
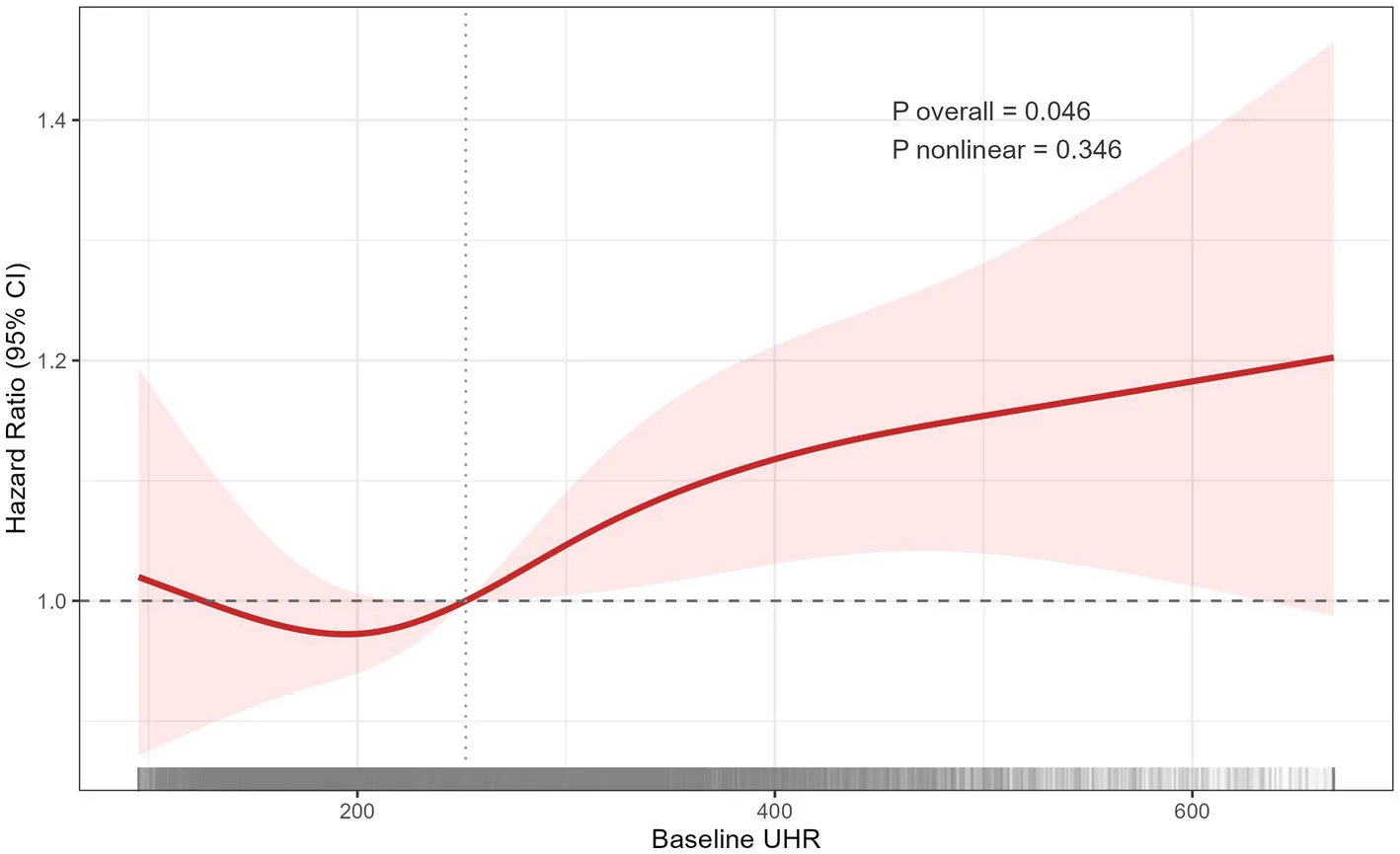
Dose–response relationship between baseline UHR and incident carotid artery lesions in the fully adjusted model. RCS Cox regression adjusted for age, sex, BMI, SBP, DBP, FBG, TG, TC, creatinine, and eGFR. The vertical dotted line indicates the median UHR (251.93), set as the reference. The shaded area represents the 95% CI. The rug plot indicates the distribution of observed UHR values.

The spline curve showed a modest dip below HR = 1.0 in the lower range of the UHR distribution (approximately UHR 150–250), with wide confidence intervals due to sparse data in that region. Above the median (UHR 251.93), the hazard ratio rose progressively with increasing UHR, reaching approximately 1.20 (95% CI approximately 1.0–1.5) at the upper extreme of the observed distribution.

### 3.4 Sex-stratified analyses

Sex-stratified Cox regression and RCS analyses are presented in Table 3 and Figure 4. Among male participants (1 SD of UHR = 114.83 units), each 1-SD increment in baseline UHR was significantly associated with a higher hazard of incident carotid artery lesions in the fully adjusted model (HR 1.07, 95% CI 1.02–1.12; *p* = 0.010). RCS analysis confirmed a statistically significant overall association (*P* overall = 0.031) with no significant non-linearity (*P* non-linear = 0.311). The male spline showed a curve starting below HR = 1.0 at low UHR values (approximately 0.80 at UHR 100, though confidence intervals were wide in this region), crossing the null at approximately UHR 300–350, and reaching a plateau at approximately HR 1.07–1.10 in the higher UHR range.

**Table 3:** Sex-stratified Cox regression and restricted cubic spline analysis of the association between baseline UHR and incident carotid artery lesions.

Variable	Male (*n* = 8,544)	Female (*n* = 8,028)	*P* for interaction
HR per 1-SD increment (95% CI)	1.07 (1.02–1.12)	0.98 (0.93–1.04)	0.261
*P*-value	0.010	0.524	–
*P* overall (RCS)	0.031	0.880	–
*P* non-linear (RCS)	0.311	0.896	–
1 SD of UHR (units)	114.83	71.08	–

Models adjusted for age, BMI, SBP, DBP, FBG, TG, TC, creatinine, and eGFR. Sex was excluded from stratified models as analyses were conducted within each sex stratum. HDL-C and UA were not included as covariates as they constitute the components of UHR. HR expressed per 1-SD increment in UHR, calculated separately within each sex stratum. RCS analysis followed the same approach as Table 2, applied to sex-specific UHR distributions, with the sex-specific median UHR as the reference value. *P* for interaction was derived from a likelihood ratio test comparing models with and without a UHR × sex interaction term.

**Figure 4:**
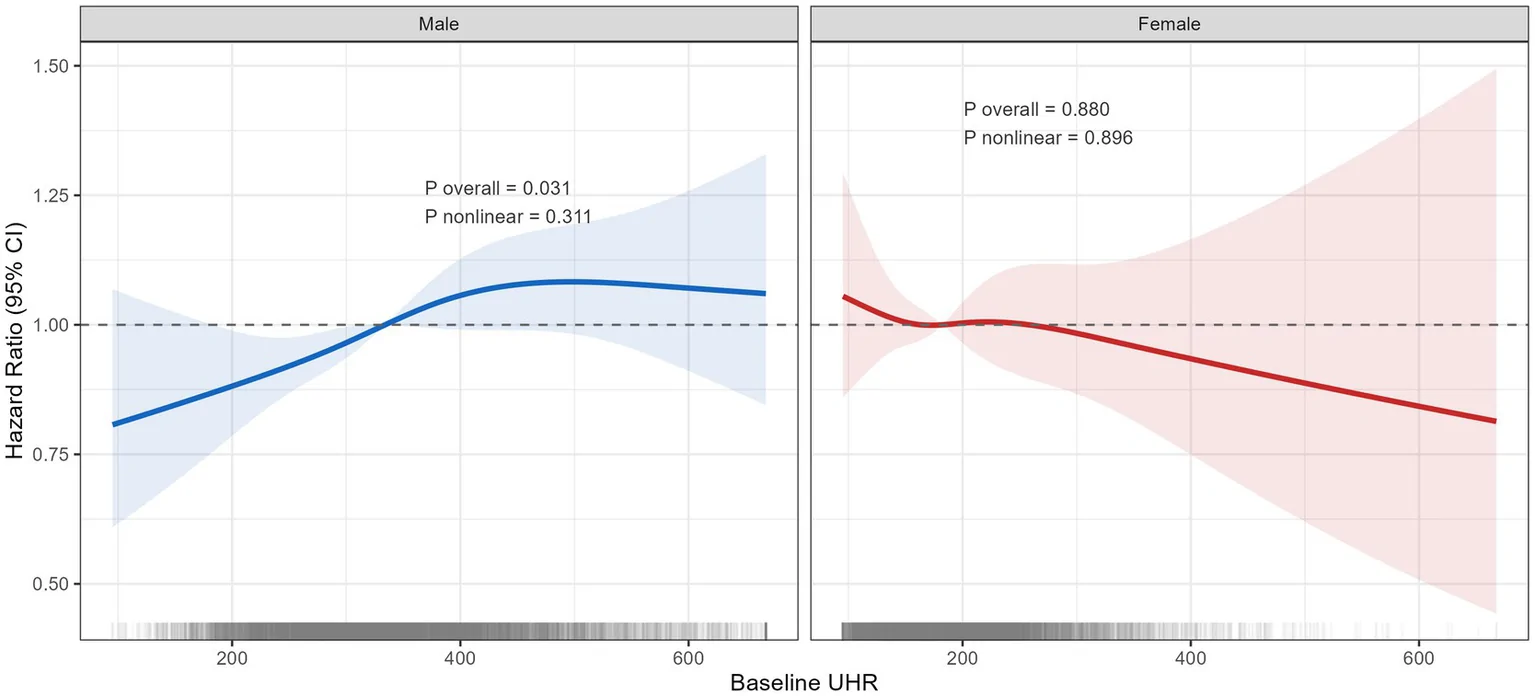
Sex-stratified dose–response relationship between baseline UHR and incident carotid artery lesions. RCS Cox regression conducted separately in male and female subgroups, adjusted for age, BMI, SBP, DBP, FBG, TG, TC, creatinine, and eGFR. The sex-specific median UHR was set as the reference value. Shaded areas represent 95% CIs. P for interaction = 0.261.

The formal test for a multiplicative interaction between UHR and sex did not reach statistical significance (*P* for interaction = 0.261). Consequently, the sex-stratified findings should be considered exploratory rather than confirmatory, and the apparent sex difference may reflect chance variation, differences in statistical power (females had narrower UHR range: 1 SD = 71.08 vs. 114.83 units in males), or unmeasured confounding. Definitive conclusions regarding sex-specific effects require prospective studies specifically designed and adequately powered to test for effect modification by sex.

Among female participants (1 SD of UHR = 71.08 units), no significant association was observed (HR 0.98, 95% CI 0.93–1.04; *p* = 0.524; *P* overall = 0.880; *P* non-linear = 0.896). The female spline showed a trend toward lower HR values with increasing UHR above the median, with the point estimate declining to approximately 0.80 at the upper end of the female UHR distribution; confidence intervals widened substantially at higher UHR values. The formal test for a multiplicative interaction between UHR and sex did not reach statistical significance (*P* for interaction = 0.261).

### 3.5 UHR longitudinal trajectory groups and incident carotid artery lesions

Associations between UHR trajectory group membership and incident carotid artery lesions are summarized in Supplementary Table 1. In the unadjusted model, both the Moderate UHR group (HR 1.27, 95% CI 1.20–1.36; *p* < 0.001) and the High UHR group (HR 1.16, 95% CI 1.06–1.28; *p* = 0.002) showed significantly higher hazard relative to the Low UHR reference group. Notably, the point estimate for the High UHR group was paradoxically lower than that of the Moderate group in the unadjusted model, a pattern that resolved after adjustment for age and sex. After adjustment for age and sex (Model 2), the HR for the High UHR group increased to 1.18 (95% CI 1.06–1.32, *p* = 0.002), while the Moderate group HR attenuated to 1.11 (95% CI 1.03–1.19, *p* = 0.008). In the fully adjusted model (Model 3), HRs for both the Moderate (HR 1.07, 95% CI 0.97–1.16; *p* = 0.163) and High UHR groups (HR 1.11, 95% CI 0.97–1.27; *p* = 0.132) no longer reached statistical significance.

### 3.6 Incremental predictive performance

To assess whether UHR improves risk prediction beyond traditional cardiovascular risk factors, we evaluated C-statistic, integrated discrimination improvement (IDI), continuous net reclassification improvement (NRI), median improvement in individual predicted event probabilities (MED), and decision curve analysis (DCA).

In the overall cohort, the addition of UHR resulted in no meaningful change in C-statistic (ΔC = 0.0003, *p* = 0.949; Supplementary Table 2). IDI was 0.000 (95% CI 0.000–0.001, *p* = 0.070), and continuous NRI was 0.054 (95% CI 0.000–0.079, *p* = 0.050). However, because the ΔC-statistic was negligible and NRI is sensitive to minor model changes, this marginal NRI significance should be interpreted with caution. MED was 0.000 (95% CI 0.000–0.001, *p* = 0.368).

In males, ΔC was similarly negligible (0.0006, *p* = 0.936). IDI was 0.001 (95% CI 0.000–0.002, *p* = 0.116), and NRI was 0.047 (95% CI − 0.001–0.078, *p* = 0.056). Notably, MED was 0.001 (95% CI 0.000–0.001, *p* = 0.008), indicating that individual predicted event probabilities changed significantly, though the magnitude of change was small (median change of 0.1%). Decision curve analysis at a 2-year time horizon demonstrated that the UHR-added model and base model yielded nearly identical net benefit across all clinically relevant risk thresholds in both the overall cohort (Supplementary Figure 3) and males (Supplementary Figure 4), with curves overlapping almost completely. This confirms that UHR does not improve population-level discrimination or clinical decision-making. Although MED was statistically significant in males (*p* = 0.008), indicating that individual predicted event probabilities changed, this small change (median 0.1%) did not translate into an appreciable improvement in clinical net benefit, as confirmed by decision curve analysis.

### 3.7 Sensitivity analyses

To assess the robustness of the primary findings, we conducted three sensitivity analyses (Supplementary Table 3). First, when Winsorization was performed at the 2.5th and 97.5th percentiles instead of the 1st and 99th percentiles, the association between baseline UHR and incident carotid artery lesions remained statistically significant (HR 1.06 per 1-SD, 95% CI 1.01–1.11, *p* = 0.019), with results materially unchanged from the primary analysis. Second, to minimize potential reverse causation, we excluded participants who developed carotid artery lesions within the first year of follow-up (*n* = 1,058 events, representing 23.7% of all events). In this restricted cohort (*n* = 15,514, with 3,396 incident events), the association remained statistically significant and slightly strengthened (HR 1.07, 95% CI 1.01–1.13, *p* = 0.014). Third, when baseline UHR was analyzed without Winsorization (using raw values), the association remained statistically significant (HR 1.06, 95% CI 1.02–1.11, *p* = 0.008). These sensitivity analyses confirm the robustness of the primary findings across different analytical specifications.

### 3.8 Internal validation

Bootstrap internal validation with 1,000 resamples confirmed the stability of the primary Cox regression estimates. The bootstrapped hazard ratio for baseline UHR (per 1-SD increment) in the fully adjusted model was 1.06 (95% bootstrap percentile CI 1.01–1.11), consistent with the original estimate of 1.06 (95% CI 1.01–1.11; Supplementary Figure 2). Only 2 of 1,000 bootstrap samples (0.2%) failed to converge, indicating excellent model stability. This confirms that the observed association is robust to resampling variation and not driven by overfitting or influential outliers.

## 4 Discussion

This retrospective cohort study demonstrated that higher baseline UHR was independently associated with an increased risk of incident carotid artery lesions following comprehensive adjustment for established cardiovascular risk factors. The dose–response relationship was broadly linear above the median UHR. The association was significant in males but absent in females, though the non-significant interaction test (*p* = 0.261) precludes definitive conclusions about sex-specific effects. UHR did not improve global discrimination or clinical decision-making beyond traditional risk factors.

Our findings extend existing cross-sectional evidence: elevated baseline UHR precedes the detection of carotid lesions in a prospective cohort. Prior studies have documented independent associations between UHR and carotid plaque in health examination cohorts and in patients with type 2 diabetes mellitus, with UHR demonstrating superior discriminative performance over UA or HDL-C alone in ROC analyses (18, 19). A global meta-analysis further identified lower HDL-C as a significant risk factor for carotid plaque, supporting the biological relevance of the HDL-C component of UHR in atherosclerotic risk (20–22). However, cross-sectional designs cannot establish temporality or distinguish prevalent from incident disease. The present study advances this literature by providing time-to-event incidence estimates using Cox regression in a general health examination population, rather than disease-specific clinical cohorts. However, the mean follow-up of 2.7 years is insufficient to distinguish true incident atherosclerosis from progression of pre-existing subclinical disease, limiting the incremental insight beyond what cross-sectional studies have already shown. The primary contributions of this study include establishing temporality through prospective design and confirming robustness through comprehensive sensitivity analyses and internal validation. Importantly, decision curve analysis demonstrated that despite statistical significance, UHR does not meaningfully improve population-level risk stratification beyond traditional factors. This negative finding tempers enthusiasm for UHR as a clinical screening biomarker.

The biological rationale for the UHR-atherosclerosis association rests on the opposing vascular effects of its components. Elevated UA has been associated with endothelial dysfunction, oxidative stress, and systemic inflammation in experimental and clinical studies, and is an established correlate of accelerated atherosclerotic progression (23–25). Reduced HDL-C independently predicts cardiovascular risk and carotid plaque burden in large-scale observational data (26–28). The UHR therefore reflects the net balance between these opposing forces as a single composite index. We did not measure oxidative stress markers or inflammatory mediators, and the mechanistic pathways linking UHR to incident carotid atherosclerosis remain uncharacterized in this cohort.

The sex-stratified findings warrant cautious interpretation. Although the association was statistically significant in males (*p* = 0.010) but not in females (*p* = 0.524), the formal interaction test was not significant (*p* = 0.261), precluding definitive conclusions about sex-specific effects. Several methodological factors could explain this apparent difference. First, the extreme sex imbalance across UHR trajectory groups (High UHR: 96.9% male; Low UHR: 79.7% female) reflects the well-established uricosuric effect of estrogen and sex differences in HDL-C metabolism (18). This creates substantial collinearity between UHR and sex, making it challenging to fully disentangle their independent effects despite statistical adjustment. However, baseline UHR modeled as a continuous variable retained independent predictive value after full adjustment (Table 2), and sex-stratified analyses confirmed a significant association in males (Table 3). Second, females had a substantially compressed UHR distribution (1 SD = 71.08 vs. 114.83 units in males), resulting in reduced statistical power. Most female participants may have had UHR values below the pro-atherogenic threshold, particularly given the protective effects of premenopausal estrogen (29–31). Third, the smaller female subgroup and correspondingly fewer incident events further limited statistical power to detect associations in females. These findings contrast with the cross-sectional report of Zhang et al. (18), who observed greater discriminative ability of UHR for carotid plaque in females. This apparent conflict may reflect fundamental differences between cross-sectional discriminative ability and prospective incident risk. Consistent with broader epidemiological evidence showing male predominance in carotid atherosclerosis (32, 33), the non-significant interaction test (*p* = 0.261) suggests that sex-specific conclusions should be interpreted cautiously. Studies with larger female subgroups and menopausal substratification are needed to definitively resolve this question.

The attenuation of trajectory-based associations to non-significance in the fully adjusted model warrants careful interpretation. Trajectory group membership captured not only sustained UHR elevation but also distinct cardiometabolic phenotype clusters. When individual metabolic components were included as covariates, the associations attenuated substantially. This suggests overlap in the variance captured by trajectory membership and traditional risk factors. Additionally, the stable between-group separation throughout follow-up suggests that baseline UHR already provided substantial information about long-term UHR exposure. Importantly, baseline UHR modeled as a continuous variable retained significant predictive value in the fully adjusted model, indicating independent prognostic information beyond traditional risk factors. These findings suggest that in health examination populations where traditional cardiometabolic risk factors are routinely monitored, baseline UHR may provide adequate risk stratification without requiring repeated measurements.

The negligible improvement in discrimination (ΔC = 0.0003 overall, 0.0006 in males) is consistent with the established difficulty of improving discrimination in models already achieving adequate performance (C-statistic 0.73–0.74). Decision curve analysis confirmed that UHR does not improve clinical net benefit across all clinically relevant risk thresholds. Although individual predicted event probabilities changed significantly in males, the magnitude of change was small (median 0.1%) and did not translate into appreciable improvement in net benefit. We reported NRI and IDI for completeness, but these metrics have well-documented limitations, including sensitivity to model specification and potential for false positive findings when discrimination improvement is negligible. We therefore prioritized more robust metrics (C-statistic and decision curve analysis) in interpreting incremental predictive performance. UHR is computed from two routine biochemical measurements at negligible marginal cost; however, these findings indicate that while it provides statistically independent prognostic information in males, it does not offer clinically meaningful incremental value for population-level risk stratification beyond traditional cardiovascular risk factors.

The absence of a J- or U-shaped association in the present study contrasts with prior reports of non-linear relationships between serum uric acid and cardiovascular risk. Several factors may explain this difference. First, prior studies reporting J- or U-shaped patterns typically examined serum uric acid alone, not the UA-to-HDL-C ratio. The ratio structure may linearize relationships that are non-linear for individual components. Second, J- and U-shaped associations are most consistently observed in clinical populations with established cardiovascular disease, heart failure, or advanced chronic kidney disease. In these populations, very low uric acid may reflect cachexia, malnutrition, or xanthine oxidase inhibitor therapy, representing reverse causation rather than a true protective effect. In contrast, our cohort represents a general health examination population with rigorous exclusion of prevalent cardiovascular disease and preserved kidney function (median eGFR 104.55 mL/min/1.73 m^2^). Low UHR in this context likely reflects favorable metabolic health (low uric acid combined with high HDL-C) rather than pathological states, which may explain the modest protective trend at low UHR values. Third, the significant non-linearity in the unadjusted model was entirely attributable to confounding by sex and resolved after adjustment. This highlights the importance of rigorous sex adjustment when examining UHR-atherosclerosis associations.

This study has several important limitations. First, the mean follow-up of 2.7 years is insufficient to capture true *de novo* atherosclerotic development, which occurs over decades. Many “incident” cases likely represent progression of pre-existing subclinical atherosclerosis rather than truly new-onset disease. Second, requiring at least two consecutive annual health examinations excluded 75% of the initial cohort, introducing substantial selection bias. The included participants likely differ from the general population in being more health-conscious, having better healthcare access, and belonging to higher socioeconomic strata. This restricts the generalizability of our findings to health examination populations rather than community-based or clinical populations. The observed association may underestimate the true effect in higher-risk populations with greater UHR variability. Third, single-center recruitment, unavailability of data on urate-lowering therapy and lipid-lowering agents, and the substantially smaller female subgroup limit the robustness and generalizability of the findings. Fourth, the large sample size enabled detection of statistically significant differences that may lack clinical significance. We therefore prioritized interpretation of hazard ratios and dose–response curves over reliance on *p*-values alone. Despite these limitations, comprehensive sensitivity analyses and bootstrap internal validation confirmed the robustness of our primary findings.

## 5 Conclusion

Higher baseline UHR was independently associated with incident carotid atherosclerotic progression in a general health examination population. The association was observed primarily in males, though sex-specific conclusions remain uncertain. UHR did not improve risk discrimination beyond traditional cardiovascular risk factors and shows limited utility for population-level screening. Future studies with larger female cohorts and longer follow-up are needed to clarify sex-specific effects and clinical applicability.

## Data Availability

The data used during the current study are available from the corresponding author upon reasonable request.
