# Supplementary material for: Uric acid-to-HDL-cholesterol ratio and incident carotid atherosclerosis: a retrospective cohort study
Source: Front Nutr. 2026 Oct 15;13:1951708. doi: 10.3389/fnut.2026.1951708 (PMC13718328; doi:10.3389/fnut.2026.1951708)
Supplement: Supplementary file 1 [file Data_Sheet_1.PDF]

Supplementary Materials

Supplementary Table 1. Association of longitudinal UHR trajectory groups with incident carotid artery lesions.

Model	Group	HR (95% CI)	P value
Model 1 (unadjusted)	Low UHR	Ref.	—
	Moderate UHR	1.27 (1.20–1.36)	<0.001
	High UHR	1.16 (1.06–1.28)	0.002
Model 2 (age- and sex-adjusted)	Low UHR	Ref.	—
	Moderate UHR	1.11 (1.03–1.19)	0.008
	High UHR	1.18 (1.06–1.32)	0.002
Model 3 (fully adjusted)	Low UHR	Ref.	—
	Moderate UHR	1.07 (0.97–1.16)	0.163
	High UHR	1.11 (0.97–1.27)	0.132

Values are hazard ratios (HRs) with 95% confidence intervals (CIs). Model 1 was unadjusted. Model 2 was adjusted for age and sex. Model 3 was adjusted for age, sex, body mass index (BMI), systolic blood pressure (SBP), diastolic blood pressure (DBP), fasting blood glucose (FBG), triglycerides (TG), total cholesterol (TC), creatinine, and estimated glomerular filtration rate (eGFR). The low-UHR trajectory group was used as the reference. UHR, uric acid-to-high-density lipoprotein cholesterol ratio.

Supplementary Table 2. Incremental predictive value of baseline UHR beyond conventional risk factors.

Metric	Overall	Male subgroup
C-statistic, base model	0.7352	0.7104
C-statistic, UHR model	0.7356	0.7110
ΔC	0.0003	0.0006
P value (ΔC)	0.949	0.936
IDI (95% CI)	0.000 (0.000–0.001)	0.001 (0.000–0.002)
P value (IDI)	0.070	0.116
Continuous NRI (95% CI)	0.054 (0.000–0.079)	0.047 (−0.001–0.078)
P value (NRI)	0.050	0.056
MED (95% CI)	0.000 (0.000–0.001)	0.001 (0.000–0.001)
P value (MED)	0.368	0.008

ΔC, difference in C-statistic; IDI, integrated discrimination improvement; NRI, net reclassification improvement; MED, median improvement in individual predicted event probabilities; CI, confidence interval. The base model included age, sex, BMI, SBP, DBP, FBG, TG, TC, creatinine, and eGFR. The UHR model included all base-model covariates plus baseline UHR as a continuous variable. Male-specific incremental predictive metrics are reported because exploratory stratified analyses suggested a possible association in males (HR 1.07, P = 0.010), though the formal interaction test was not significant (P = 0.261).

Supplementary Table 3. Sensitivity analyses of the association between baseline UHR and incident carotid artery lesions.

Analysis	HR (95% CI)	P value	Notes
Primary analysis	1.06 (1.01–1.11)	0.015	Winsorized at the 1st and 99th percentiles
Alternative winsorization (2.5th/97.5th percentiles)	1.06 (1.01–1.11)	0.019	Winsorized at the 2.5th and 97.5th percentiles
Excluding events occurring within the first year	1.07 (1.01–1.13)	0.014	Excluded 1,058 incident events occurring within the first year
No Winsorization	1.06 (1.02–1.11)	0.008	Raw UHR (no winsorization)
Bootstrap validation (1000 resamples)	1.06 (1.01–1.11)	—	Bootstrap percentile CI

HRs are reported per 1-standard deviation (SD) increase in baseline UHR from the fully adjusted Cox model, with adjustment for age, sex, BMI, SBP, DBP, FBG, TG, TC, creatinine, and eGFR. The same covariate set was used in each sensitivity analysis unless otherwise specified.

Supplementary Figure 1. Dose-response association between baseline UHR and incident carotid artery lesions across three sequential adjustment models.

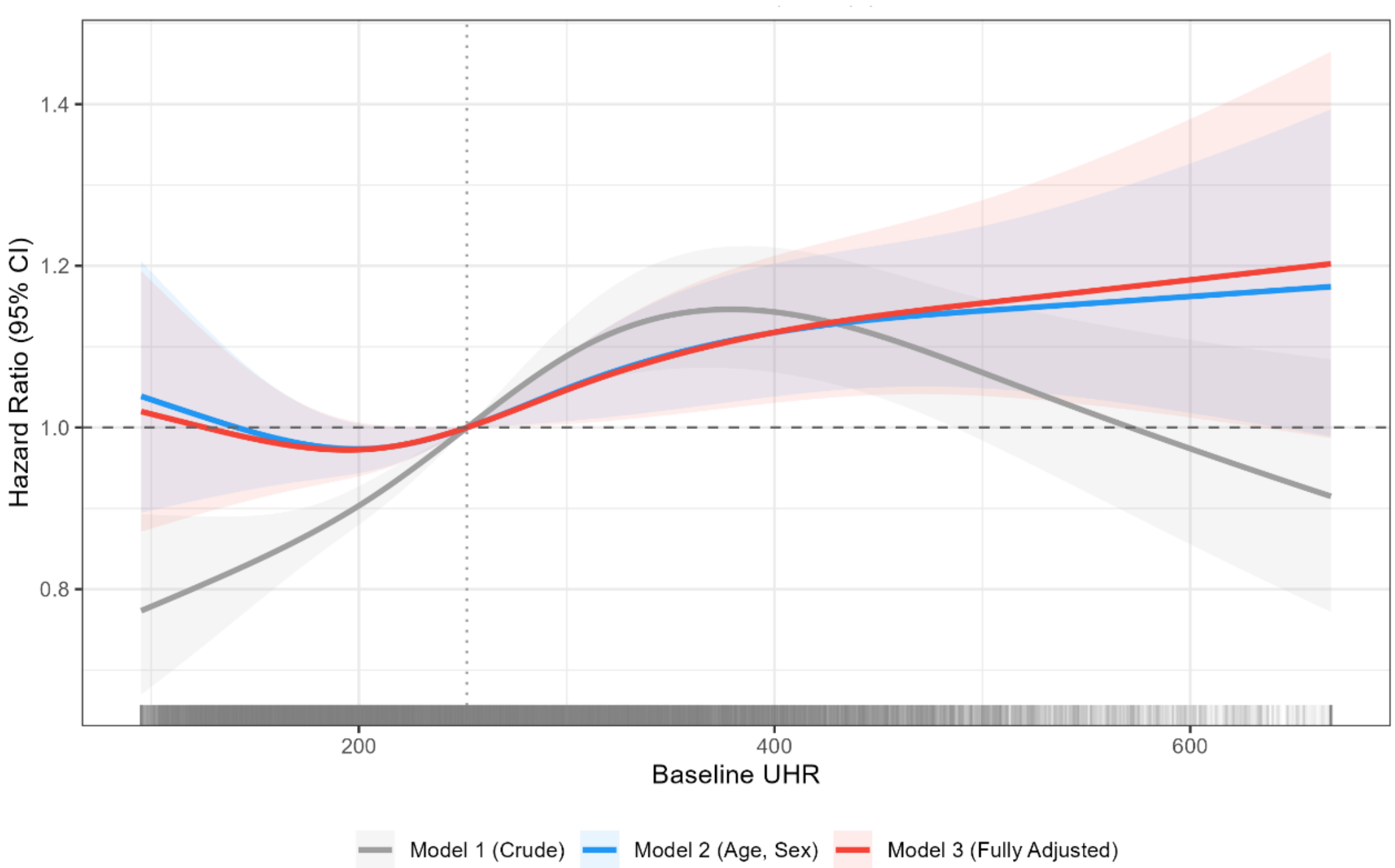

Restricted cubic spline Cox regression was performed using the median baseline UHR (251.93) as the reference value (HR = 1.00). Shaded areas represent 95% CIs. Model 1 was unadjusted; Model 2 was adjusted for age and sex; and Model 3 was adjusted for age, sex, BMI, SBP, DBP, FBG, TG, TC, creatinine, and eGFR.

Supplementary Figure 2. Bootstrap distribution of hazard ratio estimates for baseline UHR.

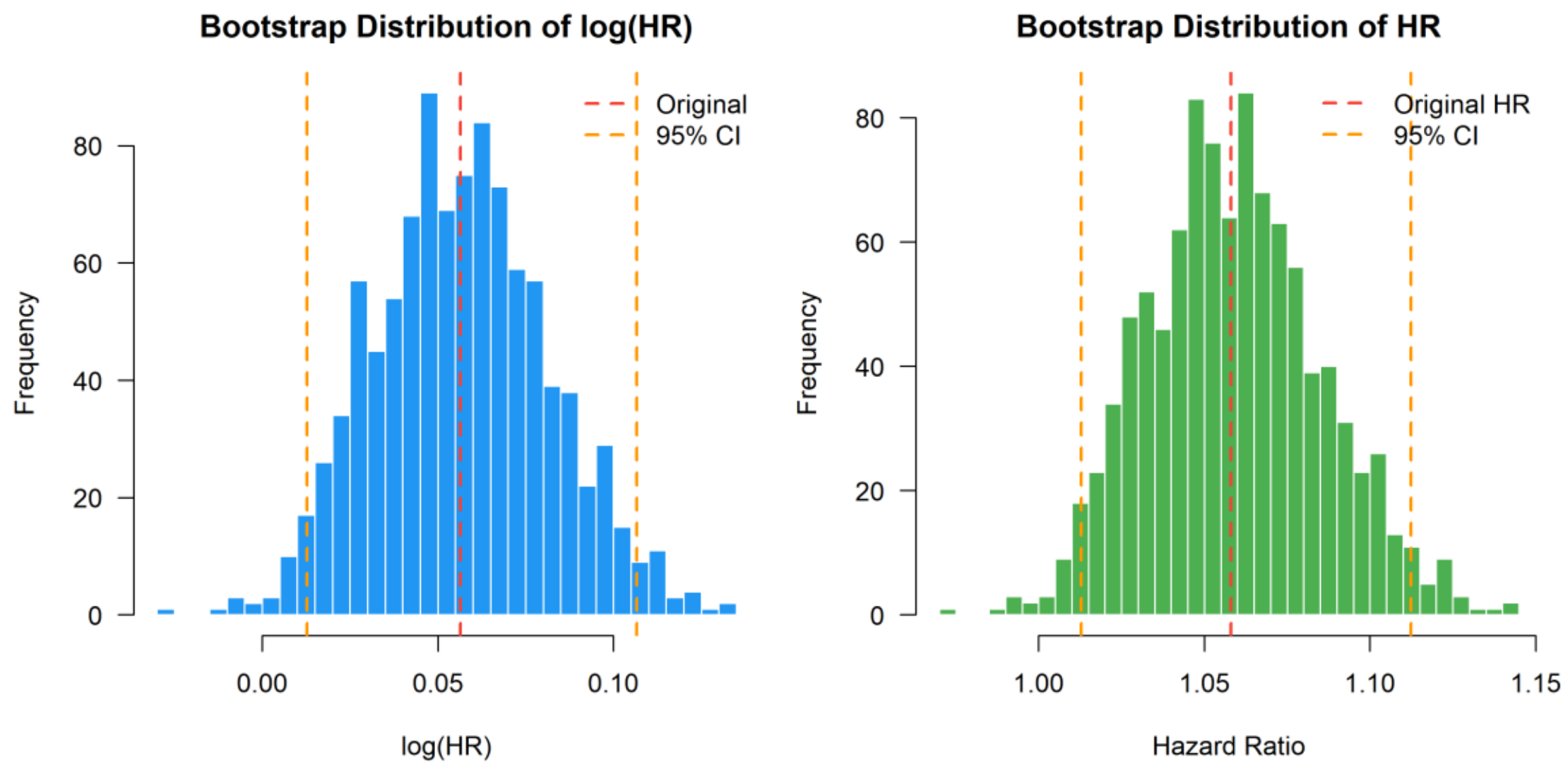

Bootstrap distributions of log(HR) and HR for baseline UHR per 1-SD increase, based on 1,000 bootstrap resamples. The red dashed line denotes the estimate from the original sample (HR = 1.06), and the orange dashed lines denote the 95% bootstrap percentile CI. The close agreement between the original and bootstrap estimates confirms the robustness of the association.

Supplementary Figure 3. Decision curve analysis in the overall cohort.

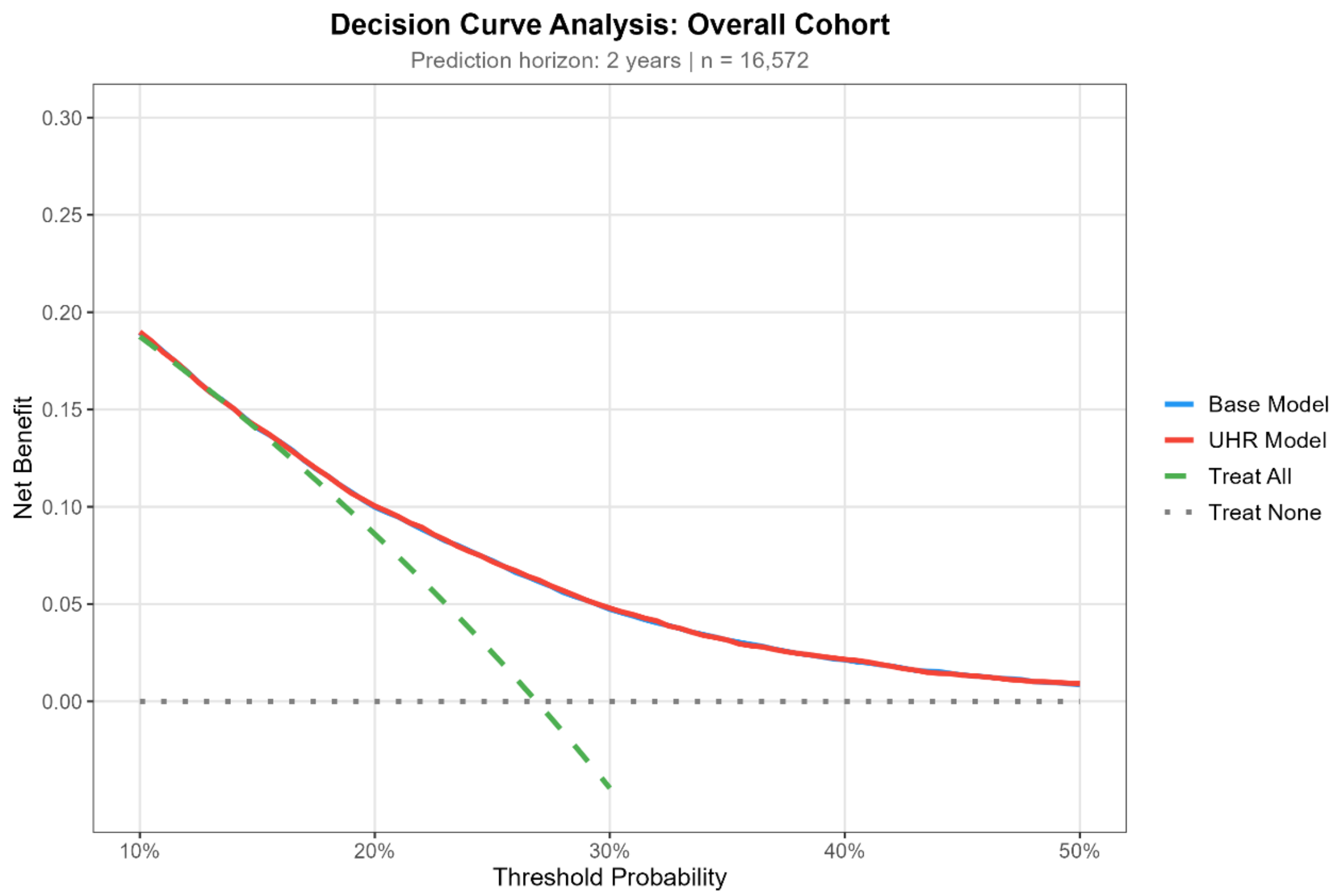

Decision curve analysis comparing the net benefit of the base model and the UHR-added model at a 2-year prediction horizon in the overall cohort (n = 16,572). The base model included age, sex, BMI, SBP, DBP, FBG, TG, TC, creatinine, and eGFR; the UHR-added model additionally included baseline UHR.

The two models showed nearly identical decision curves across threshold probabilities of 10%–50%, with curves overlapping almost completely, indicating that UHR does not improve net benefit beyond conventional risk factors.

Supplementary Figure 4. Decision curve analysis in the male subgroup.

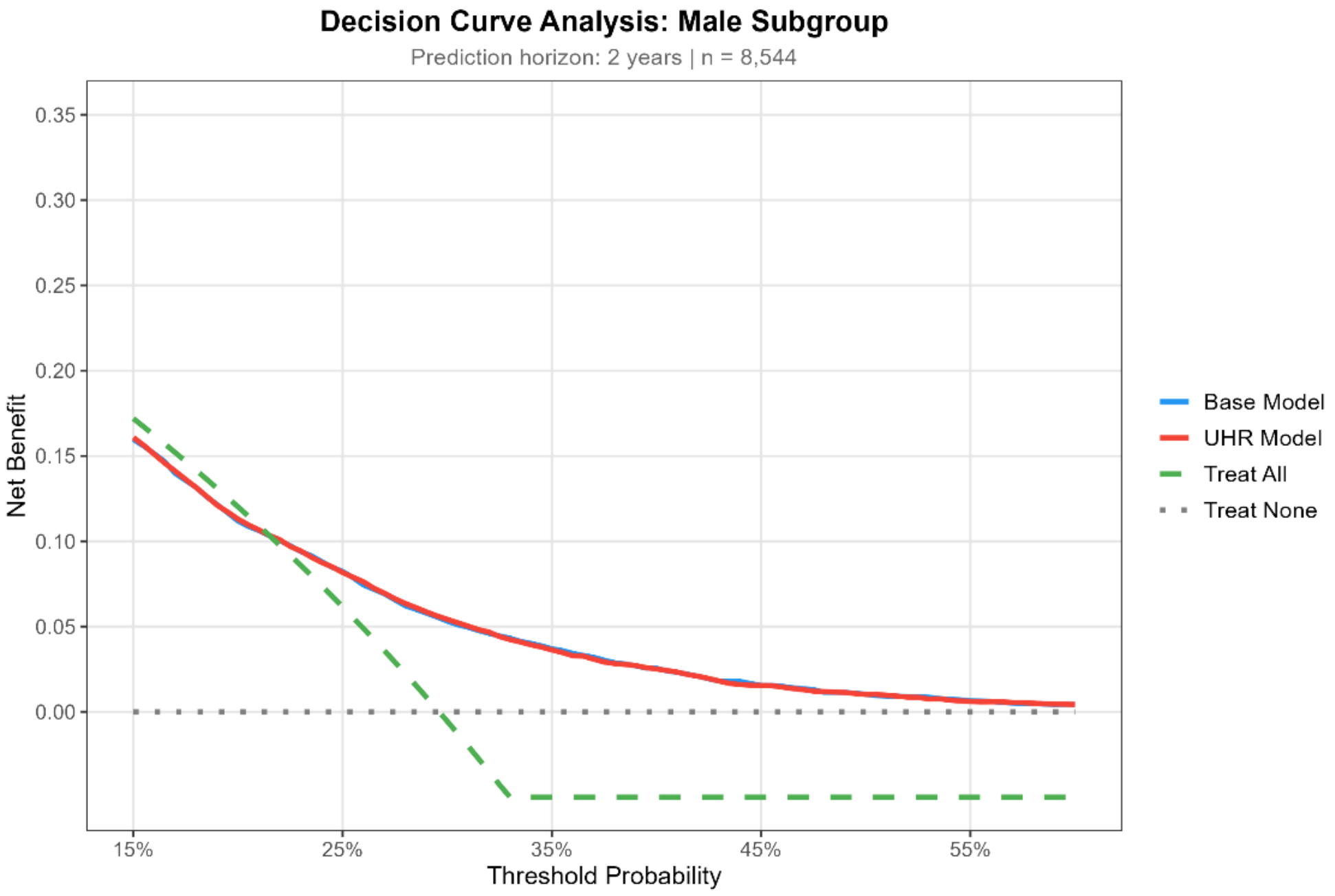

Decision curve analysis comparing the net benefit of the base model and the UHR-added model at a 2-year prediction horizon in the male subgroup (n = 8,544). The base model included age, BMI, SBP, DBP, FBG, TG, TC, creatinine, and eGFR; the UHR-added model additionally included baseline UHR. The two models showed nearly identical decision curves across threshold probabilities of 15%–60%, with curves overlapping almost completely. Despite the statistically significant MED in males (Supplementary Table 2), this small change (median 0.1%) did not translate into improved net benefit.
